# A systematic review of qualitative evidence on factors enabling and deterring uptake of HIV self-testing in Africa

**DOI:** 10.1186/s12889-019-7685-1

**Published:** 2019-10-15

**Authors:** Bernard Njau, Christopher Covin, Esther Lisasi, Damian Damian, Declare Mushi, Andrew Boulle, Catherine Mathews

**Affiliations:** 1 School of Public Health and Family Medicine, University of Cape Town, Cape Town, South Africa; 2 Kilimanjaro Christian Medical University College, Moshi, Kilimanjaro Tanzania; 3 Health Systems Research Unit, South African Medical Research Council, Cape Town, South Africa

**Keywords:** HIV, AIDS, HIV testing, HIVST, Barriers, Facilitators, Africa

## Abstract

**Background:**

More than 40% of adults in Sub-Saharan Africa are unaware of their HIV status. HIV self-testing (HIVST) is a novel approach with a potential to increase uptake of HIV testing and linkage to care for people who test HIV positive. We explored HIV stakeholder’s perceptions about factors that enable or deter the uptake of HIV self-testing and experiences of self-testing of adult users in Africa.

**Methods:**

This systematic review of qualitative evidence included articles on qualitative studies published or made available between January 1998 to February 2018 on perspectives of key stakeholders, including HIV policymakers, HIV experts, health care providers, and adult men and women (18 years and above) about factors that enable or deter the uptake of HIV self-testing and experiences of self-testing among adult users.

We searched CINAHL, MEDLINE in Pubmed, EMBASE, AJOL, PsycINFO, Social Science Citation Index (SSCI), and Web of Science for articles in English on HIVST with qualitative data from different African countries.

**Results:**

In total, 258 papers were retrieved, and only nine (9) studies conducted in 5 African countries were eligible and included in this synthesis.

Perceived facilitators of the uptake of HIVST were autonomy and self-empowerment, privacy, confidentiality, convenience, opportunity to test, including couples HIV testing, and ease of use. The perceived barriers included the cost of buying self-test kits, perceived unreliability of test results, low literacy, fear and anxiety of a positive test result, and potential psychological and social harms. HIV stakeholder’s concerns about HIVST included human right issues, lack of linkage to care, lack of face-to-face counseling, lack of regulatory and quality assurance systems, and quality of self-test kits. Actual HIVST users expressed preference of oral-fluid self-testing because of ease of use, and that it is less invasive and painless compared to finger-stick/whole blood-based HIV tests. Lack of clear instructions on how to use self-test kits, and existing different products of HIVST increases rates of user errors.

**Conclusions:**

Overcoming factors that may deter HIV testing, and HIVST, in particular, is complex and challenging, but it has important implications for HIV stakeholders, HIVST users, and public health in general. Research is warranted to explore the actual practices related to HIVST among different populations in Africa.

## Background

HIV is a serious public health burden in Africa, particularly in sub-Saharan Africa (SSA). More than 75% of HIV infected people are in Africa, and nearly half (45.7%) of newly diagnosed cases of HIV among adults are among Africans. Efforts to achieve the global target of 95–95-95 by 2030 require increased uptake of HIV testing as an entry point to HIV cascade [1, 2]. Many African countries have scaled up HIV testing services, coupled with increasingly wider availability of antiretroviral therapy (ART) [1]. More than 25% of adults in Africa do not know their HIV status, irrespective of the availability of a wider provision of HIV testing services, making access to ART less successful [1, 2]. HIV Self-Testing (HIVST) has been introduced as an innovative tool with the potential for reaching high-risk, and hard-to-reach- populations including young people with HIV testing. HIVST, which does not provide a definitive diagnosis, enable potential users to know their serostatus. Those with reactive self-test results need further confirmatory HIV testing at a health facility, following national testing algorithms [3, 4].

Currently, there are two rapid diagnostic tests for HIVST, namely: finger prick test and oraQuick test (oral test) [5–7]. The finger prick self-test prototype comprises of a bag with a test cassette, diluent vial, disinfectant wipe, compression swab, lancet, sampler stick, dressing and manufacturer’s instruction for use. In brief, a drop of blood collected by a sampler stick is placed into the test cassette, and two drops of diluent are added before reading of the results after waiting for 10 min [7].

The Ora Quick® rapid HIV 1/2 antibody test (OraSure Technologies, Bethlehem, PA, USA) is the first WHO prequalified HIVST kit [8]. The Ora Quick® rapid HIV 1/2 antibody test is a lateral-flow, immuno-chromatographic, second-generation, oral-fluid assay detecting antibodies to HIV-1 and HIV-2. The Ora Quick test kits consist of two pouches; one contains a diluent tube and the second contains the test device and instruction for use. An oral fluid swab collected using the flat-pad of the test devise from upper and lower gums is placed into a pre-filled tube of reagent for 20 min before reading the results [9, 10].

Globally, there is increasing evidence on enablers to, and barriers for HIVST reported in the literature [11–14]. Convenience, short testing and waiting time, privacy, autonomy/sense of self-empowerment, use of oral fluid instead of blood-samples, and, perceived control of one’s health choices, are examples of key motivators [3, 12–14]. The easy to use procedures for collecting the oral fluid sample and a waiting time of 20 min before getting a result favors preference for oral fluid HIV test [3, 12–14]. Various HIVST related barriers are reported in the literature from studies conducted globally. Such barriers includes lack of policies on HIVST, misperceptions on quality of the self-test kits, and perceived adverse effects associated with self-testing for HIV [15–18].

For potential users who fear needle pricks for obtaining a blood sample may find blood-based HIVST testing a barrier [15, 17]. Another barrier is the cost of buying the self-test kits. Most people in Low and Middle-income countries (LMICs), including Africa, may not afford to pay for the self-test kits, ranging from $ 4.8 to $ 40 in different settings [15, 18].

Finally, inability to read among potential users may limit the uptake of unsupervised HIVST, whereby an individual is supposed to test following the instruction for use document while testing alone in privacy [18]. In 2016, the WHO recommended HIVST as a strategy to increase universal coverage of HTS among high-risk and hard-to-reach populations, but most African countries have not introduced HIVST, because HIV policymakers have reservations about the introduction of HIVST [4]. Key concerns frequently mentioned by HIV policymakers include lack of policies and regulatory systems, quality of self-test kits, ethical and human right issues, and knowledge gaps about HIVST [4, 8, 19]. This review aimed to explore the existing qualitative evidence on the factors, which may enable or deter the uptake of HIVST among adults in Africa.

### Objectives

The objectives of the systematic review of qualitative evidence were to identify, assess and analyze the evidence from qualitative studies on HIV stakeholder’s and potential adult users’ views about factors that enable or deter the uptake of HIVST, and adult users’ experiences of HIV self-testing in Africa.

## Methods

This systematic review of qualitative evidence (i.e., qualitative evidence synthesis) used a methodology described in a previous published systematic review protocol [20].

### Search strategy

This review used a search strategy published previous by the authors [20] and a summary is presented in attached Additional file 1.

### Inclusion and exclusion criteria

Studies were eligible if qualitative research methods (interview, focus groups, observations, and review of documents) or open-ended questions in questionnaires were used to explore factors that enable or deter the uptake of HIVST and testing experiences of adult users. The populations of interest were: HIV stakeholders, such as HIV policymakers, HIV experts, health care providers, and adult users of HIV self-testing. Because of language constraints, studies not published in English were excluded. Conference abstracts were included only if they represented original qualitative data.

### Data collection

#### Extraction and management

**Fig. 1:**
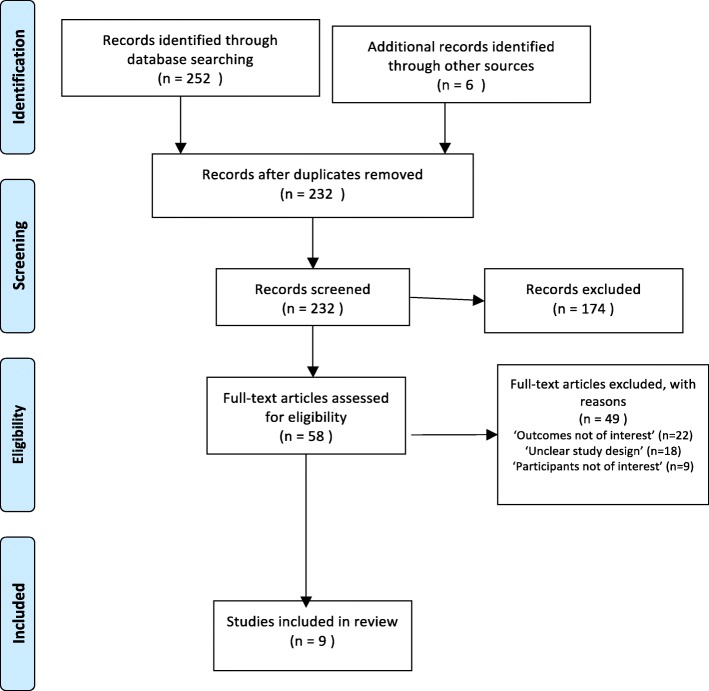
Flow diagram through different phases of the review

A pre-designed data extraction form specifically for this synthesis was used for data extraction (see Additional file 2). We extracted themes, ideas, and categories applicable to the synthesis objectives. The categories originated from views of HIV stakeholder’s views about their perceived barriers to, facilitators for the uptake of HIVST, and HIVST experiences of adult users. We obtained from the result section of each paper, the researcher’s interpretations, described in the form of themes, or ideas, or categories. We also scrutinize the discussion sections and obtained pertinent information, which was also supported with researcher’s interpretations. Additional information extracted was: the first author’s name; date of publication; language; country of study; study settings; study participants; the HIVST approaches used; theoretical or conceptual frameworks applied, and methodology of the study. Articles not meeting eligibility criteria were excluded, and those meeting the inclusion criteria were selected for full-text review (Fig. 1).

#### Quality assessment of included qualitative studies

We used the Critical Appraisal Skills Programme (CASP) quality assessment tool (see Table 2) to assess the methodological quality (or limitations) of the qualitative studies [28]. We conducted a pilot trial on three included studies to assess the feasibility of the use of the tool and to ensure the integrity of the assessment. We acknowledge that there is no gold standard approach for assessing the methodological quality (or limitations) of primary qualitative studies, but agreed that the adapted CASP checklist was a reasonable framework to assess such limitations. One reviewer applied the appraisal framework to each included study. A second reviewer checked for discrepancies. Disagreements were resolved through dialogue or by consulting a third reviewer.

The assessment of methodological quality (or limitations) was not used to exclude studies but rather to judge the relative contribution of each included study. The aim was to understand how each study contributed to the development of explanations and relationships, and as part of the assessment of confidence in each review finding.

#### Assessment of confidence in the review findings

We assessed each review finding from included qualitative studies using the GRADE-Confidence in the Evidence from Reviews of Qualitative research (GRADE-*CERQual:* the certainty of qualitative evidence) approach [29]. We used *CERQual* to transparently assess and describe how much confidence to place in the review findings. In the *CERQual* approach assessment of certainty is based on four key components:

The methodological quality of individual studies is the extent to which there are concerns about the design or conduct of the primary studies that contributed evidence to an individual review finding [29–31]. The methodological limitations of the included studies contributing to each review finding were assessed using the modified CASP tool described above.

The coherence of the review finding is an assessment of how clear and cogent (i.e. well supported or compelling) the fit is between the underlying data from the primary studies and a review finding that synthesizes that data [29, 30, 32]. The coherence of each review finding was assessed by exploring to what extent clear patterns could be identified across the data contributed by each study. Further, we sought plausible explanations if variation across studies existed.

(i) Adequacy of the data is an overall determination of the degree of richness and quantity of data contributing/or supporting a review finding [29, 30, 33]. The adequacy of the data for each review finding was assessed in terms of the “thickness” of data, the number of studies, and the stratification of countries and/or regions.
(ii) The relevance of included studies to the review question is the extent to which the body of evidence from the primary studies supporting a review question applies to the context specified in the review question [29, 30, 34]. The relevance of each review finding to the research question was assessed in terms of perspective or population, a phenomenon of interest, settings, place, intervention, and findings.

After assessing each of the four components, we reported as having: minor, moderate, serious methodological limitations; no or very minor, moderate, serious concerns about coherence; very thin or thin data, moderate rich adequacy of data, and unclear, partial, direct relevance. For the overall confidence, we used four levels to indicate the confidence of the qualitative evidence: high, moderate, low, and very low [29, 30]. Our judgments were based on an initial assumption that all review findings were ‘high confidence' and then downgraded by one, two, or three levels if there were important rather than minor concerns regarding any of the four CERQual components. The key findings, the confidence of evidence for each finding, and an explanation of the assessment of the certainty of the qualitative evidence are presented in a summary table [28].

#### Analysis and synthesis process

We conducted thematic synthesis for enablers of, and deterrents to the uptake of HIVST, and HIV self-testing experiences, using framework analysis [35–39]. The thematic analysis suit studies with a priori aims and objectives designed to directly inform policy and practice. The thematic synthesis aimed to enhance understanding of questions regarding: ‘what works for whom and in what context’, and to identify ‘barriers’ and ‘facilitators’ to the uptake of HIVST. We ‘pooled’ the results from individual primary studies by initially separating the findings, interpreting and then combining all through the identification of key themes across studies [38, 39].

Two reviewers independently coded key descriptive themes on HIV self-test experiences, enablers of, and deterrents to the uptake of HIVST. We discussed the resulting themes and sub-themes within the study team as analysis progressed to examine their relationship to the synthesis outcomes. The basic units of the review were elements of the texts reported in the ‘result’ section of each primary study included in the analysis. The text from each primary study was extracted verbatim and entered into a spreadsheet.

Familiarisation with the dataset included reading and rereading the textual data. Sections of the text were coded, with multiple codes being allocated where appropriate. The qualitative synthesis then proceeded by using the ‘descriptive themes’ to develop ‘analytical themes’, which were interpreted about the synthesis aims. During the analysis, differences or similarities were identified within emerging themes [38, 39].

This qualitative synthesis of evidence is reported following the ENTREQ statement guidelines to ensure transparency (see Additional file 3: Table S3.) [21].

#### Ethical considerations

This study did not undertake any formal data collection involving any humans or animals.

## Results

### Database search

A total of 258 papers were found across the three databases. After excluding duplicates, 232 articles remained. Out of these, 174 were excluded based on title or abstract was deemed to hold no relevance to the current synthesis based on the inclusion and exclusion criteria. We retrieved full text for the remaining 58 articles. Out of 58 full-text articles assessed for eligibility, 49 articles were excluded due to: ‘outcome not of interest’ (*n* = 22), ‘unclear study design’ (*n* = 18), and ‘participant not of interest ’ (*n* = 9). Nine (9) studies met the inclusion criteria and are included in this synthesis (see Fig. 1).

### Study characteristics

The nine (9) studies, including 397 participants included in this synthesis were conducted in five (5) countries, namely in Malawi, South Africa, Tanzania, Kenya, and Zimbabwe. The studies were conducted in 2011 (*n* = 1); 2013 (*n* = 3); 2015 (*n* = 2); 2016 (*n* = 2), and 2017(*n* = 1). Most studies used a mixed-sex sample (*n* = 6), female only (*n* = 2), and male-only (*n* = 1). Studies were conducted with actual HIVST users (adult men and women in the general population; *n* = 2), potential HIVST users (adult men and women in the general population; *n* = 3), HIV stakeholders (e.g. HIV experts, HIV policymakers, researchers, ethicists, etc.) and health care providers, (*n* = 3), and pregnant women attending antenatal clinic, and their male partners (*n* = 1). All of the included studies were published in peer-reviewed journals. In general, studies gave some description of the strategies they had used to select participants and to collect and analyze data, although these descriptions tended to be brief (Table 1).

**Table 1:** Characteristics of included studies (*n* = 9)

Author (year of study)	Country	Study aim	Study design and methods	Participants	Summary of findings
Peck et al.,(2013) [5]	Kenya, Malawi, South Africa	To evaluate the usability of a wide variety of test features suitable for HIV self-test kits.	Mixed method approach design-in-depth interviews; A framework approach	Women (*n* = 150) aged 18 years and older	Pictorial instructions, simple sample collection with integrated test components, and easy steps for interpretation of results may facilitate usability of HIVST.
Njau et al., (2011) [15]	Tanzania	To identify characteristics of HIV testing options associated with individuals’ preferences for HIV testing.	Qualitative design-in-depth interviews and focus group discussions; a note based approach.	Men (*n* = 18) and Women (*n* = 22) aged 18 years and older.	Self-test for HIV was perceived less feasible for scale-up due to the unfamiliarity of HIVST; lack of counseling and accuracy of test results were perceived barriers.
vanRooyen et al.,(2015) [21]	Kenya, Malawi, South Africa	To assess the perceptions of HIVST among stakeholders in three sub-Saharan countries.	Qualitative design-in-depth interviews; thematic analysis.	HIV policy makers, HIV experts, and health care providers (*n* = 54).	HIVST is an important complementary approach to existing conventional HIV testing services; contextual and operational evidence needed to contribute to normative WHO guidance.
Choko et al. (2016) [22]	Malawi	To explore views regarding the acceptability of offering HIV-self test kits alone or in combination with linkage intervention to ANC attendees aimed at their male partners.	Qualitative design-in-depth interviews and focus group discussions; simple descriptive content analysis.	Men (*n* = 28) and Pregnant women (*n* = 34) aged 18 years and older attending ANC	Perceived highly acceptability of woman-delivered HIVST among pregnant women attending ANC and their male partners; HIVST was not likely to lead to adverse events (i.e., IPV); conditional financial incentives may motivate male partners to link into HIV care post-HIVST.
Makusha et al., (2013) [23]	South Africa	To explore attitudes, opinions, and experiences among key stakeholders regarding HIVST in South Africa.	Qualitative design-in-depth interviews; constant comparison method	Key HIV stakeholders, including government officials, HIV experts, health care providers (*n* = 12)	HIVST has the potential to reach hard-to-reach groups, including men.
Jennings et al., (2015) [24]	Tanzania	To assess perceived cost advantages and disadvantages of using HIVST kits among infrequent and never HIV-tested urban men in Tanzania.	Mixed-method design-in-depth interviews; Inductive content analysis	Men (*n* = 23) aged 15 years and older	Financial gains and losses influence men’s decision process to HIVST; low fees or free HIVST, reduced travel time, clinical costs, and, time lost from earning income may increase the uptake of HIVST.
Kumwenda et al.,(2013) [25]	Malawi	To explore factors shaping the decision-making of cohabiting couples who opted to self-test in Blantyre, Malawi.	Qualitative design-in-depth interviews; detailed content analysis.	Men (*n* = 17), and Women (*n* = 17) aged 18 years and older	Gender roles and relationship dynamics may influence the implementation of community-based HIVST among couples.
Knight et al. (2017) [26]	South Africa	To assess the perceived usability and acceptability of HIVST among lay users using several self-test prototypes.	Mixed-method design-in-depth interviews and exit questionnaire;	Men (*n* = 23) and Women (*n* = 27) lay users aged 18 years and older in rural and peri-urban settings.	Perceived highly acceptability and readiness in the context of prototypes influenced by usability and perceived needs. Perceived easiness-to-use, privacy, autonomy, ease access, widespread availability of test kits, low or free kits, emerged as important factors influencing acceptability and desirability.
Indravudh et al., (2016) [27]	Malawi & Zimbabwe	To identify young people’s preferences for HIV-self-testing (HIVST) delivery, determine the relative strength of preferences, and explore behaviours and perceptions underlying preferences.	Mixed-method design-in-depth interviews and focus group discussions; Framework analysis	Men (*n* = 54) and Women (*n* = 68) aged 16–25 years old.	Young people believe that home-based distribution of low price self-test kits may optimize HIVST services.

### Quality assessment of included qualitative studies

The quality assessment of included studies was using standardized criteria based on the CASP tool, which examined 10 criterions. Study quality was scored according to the CASP critical score as follows: If the criterion was completely met = 2 points; criterion partially met = 1 point; and criterion not applicable/ unmet/not mentioned = 0. Finally, the study quality was classified accordingly: A total score of 20 = high quality; 16–19 = moderate quality; and ≤ 15 = low quality. None of the study findings were assessed to be of high quality, because of a lack of information regarding the relationship between researchers and participants. All nine studies were categorized as moderate quality (total score = 18–19). See Table 2.

**Table 2:** CASP critical appraisal of studies included in this review (*n* = 9)

1^st^Author(year of study)	CASP criterion ^a^										
	1	2	3	4	5	6	7	8	9	10	Total Score ^b^
Peck et al.,(2013) [5]	2	2	2	2	2	1	2	2	2	2	19
Njau et al., (2011) [15]	2	2	2	2	2	1	2	2	2	1	18
vanRooyen et al.,(2015) [21]	2	2	2	2	2	1	2	2	2	2	19
Choko et al., (2016) [22]	2	2	2	2	2	1	2	2	2	2	19
Makusha et al., (2013) [23]	2	2	2	2	2	1	2	2	2	2	19
Jennings et al., (2015) [24]	2	2	2	2	2	1	2	2	2	2	19
Kumwenda et al.,(2013) [25]	2	2	2	2	2	1	2	2	2	2	19
Knight et al.(2017) [26]	2	2	2	2	1	2	2	2	2	2	19
Indravudh et al., (2016) [27]	2	2	2	2	2	1	2	2	2	2	19

^a^CASP criterion: 1. Was there a clear statement of the aims of the research? 2. Is a qualitative methodology appropriate? 3. Was the research design appropriate to address the aims of the research? 4. Was the recruitment strategy appropriate to the aims of the research? 5. Was the data collected in a way that addressed the research issue? 6. Has the relationship between researcher and participants been adequately considered? 7. Have ethical issues been taken into consideration? 8. Was the data analysis sufficiently rigorous? 9. Is there a clear statement of findings? 10. How valuable is the research?^b^CASP critical score: a) Criterion is completely met = 2; b) criterion is partially met = 1; c) criterion not applicable, not met, or not mentioned = 0; Total score 20 = high quality; 16–19 moderate quality; ≤ 15 low quality

All of the included studies gave a clear statement of the aims of their research, using either in-depth interviews and/ or focus group discussions. None of the studies used long-term ethnographic research. Furthermore, none of the included studies discuss reflexivity (i.e. consideration of the relationship between the researchers, participants, and study settings). Most of the included studies had a description of data analysis, using different analysis strategies, such as descriptive or exploratory approaches. Most of the included studies had their findings supported by the data, except for one study with a relatively short description of the preference of HIVST among study participants. The general lack of ‘thick description’ may have been due to the study aims, or choice of methods in which the studies were conducted.

### Confidence in the findings of the review

As described in the methods section, we used the CERQual approach to assess the confidence of each review finding, grading each finding as either of high, moderate or low confidence. We assessed most of the findings as of moderate confidence because of the methodological limitations of the underlying studies. We assessed one study to be of low confidence because of concerns regarding both methodological limitations and adequacy with limited data. In this review, twenty-one (21) statements were generated and summarized into four (4) themes: potential facilitators of HIVST perceived barriers to HIVST, concerns about HIVST, and HIVST experiences. The confidences in the findings of the review are summarized in Table 3.

**Table 3:** CERQual evidence profile

Summary of review findings	Methodological limitations^a^	Coherence^b^	Adequacy^c^	Relevance^d^	CERQual assessment of confidence in the evidence	Explanation of CERQual assessment
Potential facilitators of HIVST						
1. HIV experts, HIV policymakers, and health care providers felt that the availability of HIVST would increase uptake of HIV testing, repeat testing, identifying first-time testers, and early diagnosis, leading to decreased HIV transmission in the general population [21, 23].	Minor methodological limitations (2 studies with unclear evidence of reflexivity).	High coherence (2 studies demonstrating a good fit between the review finding and the underlying data).	Moderate concerns about adequacy of data (2 studies offering thin data contributing to the review finding).	High relevance (2 studies among HIV experts and HIV stakeholders from Kenya, Malawi, and South Africa with direct relevance to the review question).	Moderate confidence	The 2 studies of moderate quality, with minor methodological limitations, no concerns about coherence, relevance and moderate concerns about adequacy of data.
2. HIV experts, HIV policy makers, health care providers, and potential HIVST users identified the potential of HIVST to decreased stigma and discrimination associated with HIV testing, and increase their motivation and uptake of self-testing [5, 21, 23–26].	Moderate methodological limitations (5 studies with unclear evidence of reflexivity and 1 study with unclear methods for data collection).	High coherence (6 studies demonstrating a good fit between the review finding and the underlying data).	No concerns about adequacy of data (6 studies offering adequately rich data contributing to the review finding).	High relevance (6 studies among HIV experts, HIV stakeholders, and potential HIVST users from Tanzania, South Africa, Malawi, and Kenya with direct relevance to the review question).	Moderate confidence	The 6 studies of moderate quality with moderate methodological limitations, no concerns about coherence, adequacy, and relevance.
3. HIV experts, HIV policy makers, health care providers, and potential HIVST users felt that HIVST will provide an opportunity to test for HIV which will circumvent facility-based barriers, leading to increased uptake of testing [5, 21, 23–26].	Moderate methodological limitations(5 studies with unclear evidence of reflexivity and 1 study with unclear methods for data collection)	High coherence (6 studies demonstrating a good fit between the review finding and the underlying data).	No concerns about adequacy of data (6studies offering adequately rich data contributing to the review finding).	High relevance (6 studies among HIV experts, HIV stakeholders, and potential HIVST from Tanzania, South Africa, Malawi, and Kenya with direct relevance to the review question).	Moderate confidence	The 6 studies of moderate quality with moderate methodological limitations, no concerns about coherence, adequacy, and relevance.
4. HIV experts, HIV policy makers, health care providers, and potential HIVST users felt that self-testing in private would increase the acceptability of HIV testing [23–27].	Moderate methodological limitations (4 studies with unclear evidence of reflexivity and 1 study with unclear methods for data collection)	High coherence (5 studies demonstrating a good fit between the review finding and the underlying data).	No concerns about adequacy of data (5 studies offering adequately rich data contributing to the review finding).	High relevance (5 studies among HIV experts, HIV stakeholders, and potential HIVST from Malawi, Zimbabwe, Tanzania, and South Africa with direct relevance to the review question).	Moderate confidence	The 5 studies of moderate quality with moderate methodological limitations, high coherence, high relevance, and no concerns about adequacy of data.
5. The autonomy to make one’s own choice of HIV testing and self-empowerment to take responsibility for HIV testing was perceived by HIV experts, HIV policy makers, health care providers, and potential HIVST users as creating a more active role in the decision-making process for HIV testing [5, 21, 23–26].	Moderate methodological limitations (5 studies with unclear evidence of reflexivity and 1 study with unclear methods for data collection).	High coherence (6 studies demonstrating a good fit between the review finding and the underlying data).	No concerns about adequacy of data (6 studies offering adequately rich data contributing to the review finding).	High relevance (6 studies among HIV experts, HIV stakeholders, and potential HIVST from Malawi, Zimbabwe, Tanzania, and South Africa with direct relevance to the review question).	Moderate confidence	The 6 studies of moderate quality with moderate methodological limitations, high coherence, high relevance, and no concerns about, adequacy of data.
6. Awareness of self-testing was perceived by HIV experts, HIV policy makers, health care providers, and potential HIVST users to be facilitated through educational campaigns to the general public using clear information about benefits of HIVST, accompanied by post-testing counseling using advanced technology (i.e., phone-based counseling) [5, 21, 23–26].	Moderate methodological limitations(5 studies with unclear evidence of reflexivity and 1 study with unclear methods for data collection).	High coherence (6 studies demonstrating a good fit between the review finding and the underlying data).	No concerns about adequacy of data (6 studies offering adequately rich data contributing to the review finding).	High relevance (6 studies among HIV experts, HIV stakeholders, and potential HIVST from Malawi, Zimbabwe, Tanzania, and South Africa with direct relevance to the review question).	Moderate confidence	The 6 studies of moderate quality with moderate methodological limitations, high coherence, high relevance, and no concerns about adequacy of data.
7. HIV experts, HIV policy makers, health care providers, and potential HIVST users reported that the convenience of self-testing in privacy brings testing services closer to users. They believed this would attenuate traveling costs, waiting time, and save time for other income-generating activities [23–27].	Moderate methodological limitations (4 studies with unclear evidence of reflexivity and 1 study with unclear methods for data collection).	High coherence (5 studies demonstrating a good fit between the review finding and the underlying data).	No concerns about adequacy of data (5 studies offering adequately rich data contributing to the review finding).	High relevance (5 studies among HIV experts, HIV stakeholders, and potential HIVST from Malawi, Zimbabwe, Tanzania, and South Africa with direct relevance to the review question).	Moderate confidence	The 5 studies of moderate quality with moderate methodological limitations, high coherence, high relevance, and no concerns about adequacy of data.
8. Potential HIVST users believed that HIVST might increase the couple’s HIV testing through face-to-face communication, that could facilitate the disclosure of HIV serostatus, and may reduce gender-based violence related to HIV positive results [22, 25].	Minor methodological limitations (2 studies with unclear evidence of reflexivity).	High coherence (2 studies demonstrating a good fit between the review finding and the underlying data).	Moderate concerns about adequacy of data (2 studies offering thin data contributing to the review finding).	Minor concerns about relevance (2 studies among potential HIVST users from Malawi with partial relevance to the review question).	Moderate confidence	The 2 studies of moderate quality, with minor methodological limitations, high coherence, moderate concern about adequacy of data, and minor concerns about relevance.
Potential barriers to HIVST:						
9. Potential HIVST users perceived that they might fail to afford HIVST kits because of concerns about the cost of buying the self-testing kits [23, 27].	Minor methodological limitations (3 studies with unclear evidence of reflexivity).	High coherence (3 studies demonstrating a good fit between the review finding and the underlying data).	Minor concerns about adequacy of data (3 studies offering moderately rich data contributing to the review finding).	Minor concerns about relevance (3 studies among potential HIVST users from Malawi, Zimbabwe, Tanzania, and South Africa with partial relevance to the review question).	Moderate confidence	The 3 studies of moderate quality, with minor methodological limitations, high coherence, and minor concerns about adequacy of data and relevance.
10. HIV experts, HIV policymakers, and health care providers believed that the type of distribution points for delivery of self-test kits might hinder the uptake of HIVST kits. Having a variety of distribution points for delivery of self-test kits that would ensure privacy and confidentiality were perceived to increase the uptake of HIVST kits [21, 23].	Minor methodological limitations (2 studies with unclear evidence of reflexivity).	High coherence (2 studies demonstrating a good fit between the review finding and the underlying data).	Moderate concerns about adequacy of data (2 studies offering thin data contributing to the review finding).	High relevance (2 studies among HIV experts and HIV stakeholders from Kenya, Malawi, and South Africa with direct relevance to the review question).	Moderate confidence	The 2 studies of moderate quality, with minor methodological limitations, high coherence, high relevance, and moderate concerns about adequacy of data.
11. Some potential HIVST users perceived that the inability of potential clients to read, particularly in rural settings, might hinder uptake of HIVST kits [15].	Moderate methodological limitation (1 study with unclear evidence of reflexivity and insufficiently rigorous data analysis).	Coherence could not be assessed as only 1 contributing study.	Serious concerns about adequacy of data (1 study offering very thin data contributing to the review finding).	Minor concerns about relevance (1 study among potential HIVST users from Tanzania with partial relevance to the review question).	Low confidence	One study of low quality, with moderate methodological limitations, unclear coherence, minor concerns about relevance and serious concerns about adequacy of data.
12. The fear and anxiety of receiving a positive test result were perceived by potential HIVST users as a barrier to uptake of HIVST kits. Buying HIVST kits was compared with buying death or poison for committing suicide [24].	Minor methodological limitation (1 study with unclear evidence of reflexivity).	Coherence could not be assessed as only 1 contributing study.	Minor concerns about adequacy of data (1 study offering moderately rich data contributing to the review finding).	Minor concerns about relevance (1 study among potential HIVST users from Tanzania with partial relevance to the review question).	Moderate confidence	One study of moderate quality, with minor methodological limitations, unclear coherence, high relevance, and minor concerns about adequacy.
13. Potential HIVST users were concerned that the oral-fluid self-test kits may fail to accurately test for HIV because they believed HIV is present in the blood only. Some potential HIVST users expressed their fear about misinterpretation of the self-test results when alone [5, 15, 24].	Moderate methodological limitation (3 studies with unclear evidence of reflexivity and 1 study with insufficiently rigorous data analysis).	High coherence (4 studies demonstrating a good fit between the review finding and the underlying data).	Minor concerns about adequacy of data (1 study offering thin data, and 3 studies offering adequately rich data contributing to the review finding).	Minor concerns about relevance (4 studies among potential HIVST users from Tanzania, Kenya, Malawi, and South Africa with partial relevance to the review question).	Moderate confidence	The 4 studies of moderate quality, with moderate methodological limitations, no concerns about coherence, relevance, and minor concerns about adequacy.
Concerns about HIVST:						
14. HIV experts, HIV policymakers, and health care providers expressed concerns about human rights issues related to HIVST. They believed that HIVST was ethical as it provides more freedom, choices and options, and power to individuals to test for HIV. However, some HIV experts cautioned that HIVST might be unethical if it increases vulnerabilities such as forced or coerced testing [21, 23].	Minor methodological limitations (2 studies with unclear evidence of reflexivity).	High coherence (2 studies demonstrating a good fit between the review finding and the underlying data).	Minor concerns about adequacy of data (2 studies offering moderately rich data contributing to the review finding).	High relevance (2 studies among HIV experts and HIV stakeholders from Kenya, Malawi, and South Africa with direct relevance to the review question).	Moderate confidence	The 2 studies of moderate quality, with minor methodological limitations, high coherence, high relevance, and minor concerns about adequacy of data.
15. HIV experts, HIV policymakers, and health care providers believed that linkage of care was an important component and inextricably linked to pre and post-test counseling. They also pointed out that a follow-up confirmatory laboratory test after a positive self-test might facilitate linkage to HIV care, treatment, and support [21, 23, 24, 27].	Moderate methodological limitations (4 studies with unclear evidence of reflexivity and 1 study with unclear methods for data collection).	High coherence (5 studies demonstrating a good fit between the review finding and the underlying data).	No concerns about adequacy of data (5 studies offering adequately rich data contributing to the review finding).	High relevance (5 studies among HIV experts and HIV stakeholders from Malawi, Zimbabwe, Tanzania, Kenya, and South Africa with direct relevance to the review question).	Moderate confidence	The 5 studies of moderate quality, with moderate methodological limitations, high coherence, high relevance, and no concerns about adequacy of data.
16. HIV experts, HIV policymakers, and health care providers expressed concerns about the absence of face-to-face HIV counseling. Lack of counseling was perceived as a key limitation of HIVST and may increase the risk of psychopathic tendencies, suicidal ideation, and coercion [21, 23].	Minor methodological limitations (2 studies with unclear evidence of reflexivity).	High coherence (2 studies demonstrating a good fit between the review finding and the underlying data).	Minor concerns about adequacy of data (2 studies offering moderately rich data contributing to the review finding).	High relevance (2 studies among HIV experts and HIV stakeholders from South Africa, Kenya, and Malawi with direct relevance to the review question).	Moderate confidence	The 2 studies of moderate quality, with minor methodological limitations, high coherence, high relevance, and minor concerns about adequacy of data.
17. Lack of effective regulation of medicines and laboratory test such as rapid HIV tests was perceived by HIV experts, HIV policymakers, and health care providers, as a major concern about quality assurance for HIVST kits. They believed that state regulation was an essential requirement to achieve quality assurance and protect users from fake/defective HIVST kits [21, 23].	Minor methodological limitations (2 studies with unclear evidence of reflexivity).	High coherence (2 studies demonstrating a good fit between the review finding and the underlying data).	Minor concerns about adequacy of data (2 studies offering moderately rich data contributing to the review finding).	High relevance (2 studies among HIV experts and HIV stakeholders from South Africa, Kenya, and Malawi with direct relevance to the review question).	Moderate confidence	The 2 studies of moderate quality, with minor methodological limitations, high coherence, high relevance, and minor concerns about adequacy of data.
18. HIV experts, HIV policy makers, health care providers, and potential HIVST users expressed fear of low quality of HIVST kits, because of their previous experiences with fake medical equipment, and false advertisements because of a lack of or poor quality assurance measures [15, 23, 26].	Moderate methodological limitations (3 studies with unclear evidence of reflexivity and 1 study with insufficiently rigorous data analysis).	High coherence (4 studies demonstrating a good fit between the review finding and the underlying data).	Moderate concerns about adequacy of data (1 study offering thin data, and 2 studies offering moderately rich data contributing to the review finding).	High relevance (4 studies among HIV experts, HIV stakeholders and potential HIVST users from Malawi, Zimbabwe, Tanzania, and South Africa with direct relevance to the review question).	Moderate confidence	The 4 studies of moderate quality, with moderate methodological limitations, high coherence, high relevance, and moderate concerns about adequacy of data.
HIV Self-testing experiences:						
19. Some HIVST users believed that HIVST creates an opportunity for previous ART users to re-start treatment, after stopping using ART because of the negative attitudes of some health care providers [5, 25].	Minor methodological limitations (2 studies with unclear evidence of reflexivity).	High coherence (2 studies demonstrating a good fit between the review finding and the underlying data).	Minor concerns about adequacy of data (2 studies offering moderately rich data contributing to the review finding).	High relevance (2 studies among actual HIVST users from Malawi, Kenya, and South Africa with direct relevance to the review question).	Moderate confidence	The 2 studies of moderate quality, with minor methodological limitations, high coherence, high relevance, and minor concerns about adequacy of data.
20. Most HIVST users felt self-test kits were easy to use, with most preferring oral-fluid based tests to finger stick/whole blood-based tests because they do not use a needle prick [5, 25].	Minor methodological limitations (2 studies with unclear evidence of reflexivity).	High coherence (2 studies demonstrating a good fit between the review finding and the underlying data).	Minor concerns about adequacy of data (2 studies offering moderately rich data contributing to the review finding).	High relevance (2 studies among actual HIVST users from Malawi, Kenya, and South Africa with direct relevance to the review question).	Moderate confidence	The 2 studies of moderate quality, with minor methodological limitations, high coherence, high relevance, and minor concerns about adequacy of data.
21. Some HIVST users expressed confusion about how to use the self-test kits because of the lack of clear instructions on some steps on how to use the kits, leading to user errors and poor accuracy of test results [5, 25].	Minor methodological limitations (2 studies with unclear evidence of reflexivity).	High coherence (2 studies demonstrating a good fit between the review finding and the underlying data).	Minor concerns about adequacy of data (2 studies offering moderately rich data contributing to the review finding).	High relevance (2 studies among actual HIVST users from Malawi, Kenya, and South Africa with direct relevance to the review question).	Moderate confidence	The 2 studies of moderate quality, with minor methodological limitations, high coherence, high relevance, and minor concerns about adequacy of data.

A summary of the review findings from the qualitative synthesis are presented here, with the relevant studies contributing to each review finding. The confidence in the evidence refers to the overall CERQual assessment of methodological limitations of included studies, relevance, adequacy, and coherence, and is rated as high, moderate, or low. The explanation of the assessment of the confidence in the evidence provides a brief assessment of each CERQual domain to support the overall CERQual assessment

^a^ When assessing methodological limitations, we consider: the 10 CASP criterion to elucidate minor/moderate/serious methodological limitations

^b^ When assessing coherence, we consider: clear and consistent patterns across primary studies and the review finding, and/or convincing explanations for the patterns of evidence in the underlying studies, and for existing variation across studies to elucidate no or very minor/moderate/serious coherence

^c^ When assessing adequacy of data, we consider: thickness of data, the number of studies, types/ number of participants, types/range of methods used across individual studies, stratification of countries and /or regions to elucidate thin / moderate/ very rich adequacy of data

^d^ When assessing relevance, we consider: phenomenon of interest, population, setting, place, intervention, and findings to elucidate partial/ indirect/unsure relevance

### Potential facilitators of HIVST

#### Availability of HIVST

The availability of HIVST was perceived by HIV experts, HIV policymakers, and health care providers as a factor that would increase uptake of HIV testing, enable repeat testing, identifying first-time testers, and early diagnosis, leading to the linkage of care and treatment.

*“We need to look at all avenues so that people can access testing services at the moment [... .], we think that the availability of self-testing in this country is going to help us to achieve that target (80%), [... .]. The more clients repeat testing the more we can identify first-time testers, and early diagnosis, and more we can link to care and treatment. “ [HIV policymaker]* [22].

#### Stigma and discrimination associated with HIV testing

Furthermore, potential HIVST users identified the potential of HIVST to decrease stigma and discrimination associated with HIV testing, which will motivate men to uptake HIVST: *Men would accept [...] they would say, “aaah, why should the doctor test me? Aaah, it’s better to be the first to know my HIV status.” You would feel shy when meeting the doctor who knows that you are HIV positive* [Male, IDI] [23].

Most participants agreed that HIVST provides an opportunity to test for HIV and to circumvent facility-based barriers, leading to increased uptake of self-testing: *Others have said it [HIVST] will alleviate the facility-related barriers-long waiting time, long queues, visibility by going to a centre or a mobile clinic. [Health care provider]* [24].

#### HIVST and confidentiality of HIV test results

The possibility of HIVST to increase the confidentiality of HIV test results compared to conventional HIV testing approaches (i.e., voluntary counseling and testing, provider-initiated counseling and testing, mobile counseling and testing, etc.) were cited by participants.

*“So I think the benefits of HIVST are pure confidentiality, if I can own the process myself, you know I would have that confidential aspect of HIV test results … ” [HIV policy maker]* [24].

#### Perceived autonomy and self-empowerment

HIV experts, HIV policymakers, health care providers, and potential HIVST users perceived that the autonomy to make one’s own choice of HIV testing method and self- empowerment to take responsibility of one’s life, including sexual health is a potential facilitator to the uptake of HIVST. They believed that the perceived autonomy and self-empowerment would create a more active role of an individual in managing own health and decision-making process for HIV testing:

” … *the self- empowerment to take responsibility for my life because if I can go as far as to decide that: “You know what, I need to be testing myself at this level”, it means I am taking responsibility for my sexual health, [. .] [and] I am going to think about it in light of how I manage my life [HIV expert]* [24].

#### Perceived convenience of self-testing

Further, the convenience of self-testing (i.e., at home and/or any private place) in privacy was perceived as a potential facilitator. Participants believed that HIVST brings testing services closer to users, and would attenuate traveling costs, waiting time at health facilities, and save time for other income-generating activities, which will encourage uptake of HIV testing.

As a 28-year-old male participant explained:

*“It is different from making the process of going to the clinic. Therefore, the number of people going to the clinic will decrease. And your daily budget, which you reserve, you will be able to buy the instrument because when you go to test at the clinic you incur costs like bus fare, eating and staying in queues. So those costs are reduced a bit “ [28- year-old men, Non-tester]* [25].

#### Couple’ HIVST and disclosure of HIV serostatus

HIV policymakers felt that HIVST may provide an opportunity for couple’s to talk before performing self-testing. They believed the face-to-face communication could facilitate the disclosure of HIV serostatus, and hence reduce gender-based violence related to HIV positive results.

*“[.*. *.] I see (HIVST) increasing couples’ talking, and in a way, we would probably reduce GBV [gender-based violence] because sometimes that’s the problem. When one goes for a test and the other doesn’t know and then the other one does find out, it is always detrimental” [HIV policy maker]* [22].

### Potential barriers to HIVST

#### Affordability of self-test kits kits

Affordability of self-test kits was a recurring theme across the studies in this review. Potential HIVST users mentioned that the high cost of buying self-testing kits might deter the uptake of HIV testing.

As a 28-year-old male participant explained:

*“That's why I said that if it is sold at a lower price like from 15,000 to 20,000 [Tanzanian shillings ~$6.91 to $9.20] people will be able to buy it. But, if it will be sold at a higher price like at 30,000 to 40,000[Tanzanian shillings~$13.80 to $18.40], others will fail to buy it – as someone may have the ability to buy it, but says why should I buy it? But at a lower price, a person can buy it ” [28-year-old-men, Non-tester]* [25].

#### Perceived unreliability of self-test results

There was a commonly discussed belief among potential HIVST users on the unreliability of self-test results. They expressed their fear that the self-test kits may fail to accurately test for HIV. This fear was based on their misconceptions about the presence of HIV in the blood sample, and the misinterpretation of results when testing alone.

*“So many people are not going for HIV test not because of the fear of the unknown but the fear that self-testing may fail to test accurately for HIV. Many people believe that HIV is in the blood … so taking a sample from the mouth to test for HIV and not a blood sample is the main reason for the fear”[Female Non-tester, IDI]* [16].

#### Low literacy and HIVST

Concern about low literacy particularly among people residing in rural settings was perceived as a potential barrier. Potential HIVST users believed that the inability to read might negatively influence the uptake of HIVST. *“Most people, particularly in the rural areas are illiterate; they can’t read even a newspaper. How can they be able to read and follow the instructions of how to use the HIV self- testing kits?” [Male tester, IDI]* [16].

#### Fear of a positive test result

One recurring theme across studies included in this synthesis was the fear and anxiety of receiving a positive test result. For example, buying of self-test kits was compared with buying death or committing suicide. As a 26-year-old male participant explained:

*“It is similar to buying death. It is like someone going to buy poison for committing suicide! So, I do not know whether the poison is right or wrong. The point of buying it is, like I said, buying my death. I mean just do not sell it. As none will buy it. If it is sold, it will be hard for someone to decide to go buy it. Trust me. You will go buy your death, I tell you” [26-year-old-men, Non-tester]* [25].

### Concerns about HIVST

HIV experts, HIV policymakers, health care providers, and potential HIVST users expressed concerns related to HIVST. Such concerns include human rights issues, lack of linkage to HIV care, and treatment, lack of face-to-face counseling, lack of regulatory and quality assurance systems, and quality of self-test kits.

#### Human rights issues

Human rights issues reported by most participants were based on how ethical is HIVST***.*** Most participants considered HIVST ethical if it would provide more freedom, choices, and options, and empower individuals to test for HIV. However, HIVST may be unethical if it will increase HIVST users vulnerabilities (i.e., coerced or forced) testing, or used to limit their freedom and rights.

*“[ … ] I can see lots of reluctance on the part of human rights people [...] It’s more the human rights people, an instinct around coerced or forced testing. It’s always about protecting the tiny percentage of people who are going to be abused.” [HIV policymaker, South Africa]* [22].

#### Lack of linkage to HIV care and treatment

Another major concern expressed by HIV experts, HIV policymakers, and health care providers was the lack of linkage to HIV care and treatment following a positive HIV result. They generally agreed that linkage to HIV care, and treatment, is an important component of HIVST and inextricably linked to counseling.

Additionally, they argued that HIVST should be regarded as a screening rather than a testing tool and put emphasis that a positive result needs to be followed by a confirmatory laboratory test at the health facility, which might facilitate linkage to HIV care, and treatment. *“[.*. *.], there need to be clear instructions on how to get into care, what needs to be done if you test positive and if there could be a reliable helpline to call, that would be ideal so that people could seek confirmatory laboratory test at the health facility … [HIV expert]* [22].

#### Lack of face-to-face counseling

Another concern about HIVST expressed by most participants was the lack of face-to-face counseling. They viewed counseling an essential component, which is missing in HIVST. From their perspective, lack of face-to-face counseling may increase the risk of psychopathic tendencies, suicidal ideation, and coercion:

*“Without adequate pre & post-test counseling, a reactive self-test result can lead to suicides, or murder, while some psychopaths may decide to embark on a ‘revenge' vendetta by hiding their status and seeking opportunities for unprotected sex. Also, a negative result may encourage the individual to engage in irresponsible sexual activities”* [HIV policy maker] [24].

#### Lack of effective regulation of HIVST

HIV experts, HIV policymakers, and health care providers perceived that lack of effective regulation of medicines and laboratory tests might jeopardize the uptake of HIVST because it would affect the quality assurance for self-test kits. Most agreed that regulatory and a quality assurance framework was essential for the uptake of HIVST.

*[ … ] several issues in terms of laws and policies on medicines and related medical supplies need to be addressed. [HIV expert]* [24].

#### Perception about the low quality of self-test kits

Beliefs about individual perceptions, and previous experiences with fake medical equipment or low quality of self-test kits, and false advertisements were perceived as a major concern in this study. Most participants expressed their fear of the low quality of self-test kits and false advertisements because of lack of poor quality assurance measures, which might undermine the uptake of HIVST:

*[ … ] How will we ensure quality assurance and ensure that the manufacturers are not false advertising? How will we ensure that the self-tests are manufactured by an accredited facility? All these issues need to be addressed to provide a good regulatory system” [HIV policy maker]* [24].

### HIV self-testing experiences

HIV self-test users believed that the availability of HIVST creates an opportunity for previous ART users to re-start treatment [40].

As explained by a female HIV positive participant:

“*I was on ARVs[...], but[...] I stopped[....] I wanted to start again but was shouted at the hospital because I did not remember my number. This [HIVST] was a better way of re-starting taking ARVs” [Female, HIV positive, Discordant]* [40].

HIV self-test users believed that uptake of HIVST could be influenced by the ease of use of self-testing kits particularly with oral fluid-based HIV compared to finger stick/ whole blood-based HIV testing. Further, most users expressed preference of oral fluid-based HIV to finger stick/whole blood-based HIV. While most users reported ease of use of self-test kits, user errors are not uncommon among self-testers. Few reported confusion on how to use self-test kits, because of lacking clear instructions on some steps on how to use the kits.

*“[The step] was a bit confusing, because at first, I didn't know if I should remove it [cap] on the test or pour over it. No instructions were available for that step. Even the picture doesn’t show.” [Male tester, South Africa]* [6].

Finally, some self-testers reported their concern about the misinterpretation of test results because of different products of HIVST with different instructions on how to interpret results to increase rates of wrong interpretations of test results.

As explained by a female HIVST user:

*“Firstly we all know that if there are two lines it means it is positive so here there are two lines and they say it is invalid, for a villager they cannot understand this, it doesn't matter where the lines are but as long as there are two lines to many positive people, so they better look into that.” [Female tester, Malawi]* [6].

## Discussion

Overall, this synthesis highlighted a broad range of qualitative evidence on potential facilitators for and perceived barriers of uptake of HIVST from HIV experts, HIV policy-makers, health care providers, and self-testing experiences of adult users in Africa.

The findings of this synthesis are important for understanding the wider array of factors that may enable or deter the uptake of HIVST, and HIVST experiences of adult users in Africa, and how they could be integrated into the broader HIV testing services. Our findings have implications for future studies assessing the feasibility of HIVST in Africa and provide valuable information for HIV stakeholders, and interventionists to consider as they develop policy, and/ or evaluate HIVST interventions.

Commonly cited potential facilitators of HIVST across the literature such as availability of HIVST, privacy & confidentiality, convenience, and disclosure to serostatus, [25, 26, 41–43] ability of HIVST to decrease stigma and discrimination, potential of HIVST to circumvent facility-based barriers, increase confidentiality of HIV test results after self-testing, and, perceived autonomy and self-empowerment in decision-making to test were also found amongst participants in this synthesis [22, 24, 44].

In this synthesis, high costs of self-test kits, the unreliability of self-testing results, low literacy, and fear and anxiety of positive test results, may mitigate effective HIVST in different settings in Africa. These findings are consistent with existing literature on potential barriers to HIVST globally [41, 44, 45]. For example, there are mixed views regarding the cost of self-test kits, across sub-regions of Africa. Since most countries in Africa are resource-poor, most participants felt that HIVST should be free of charge subsidized by the government, as the current conventional HIV testing approaches [25, 43]. While in other settings, some participants were willing to pay for self-test kits, only if they were ensured of privacy and confidentiality at distribution points for delivery of self-test kits [22, 41, 43, 46]. However, there is a gap in the literature on how to achieve free of charge self-test kits in the for-profit context and calls for empirical research to fill this gap [27].

The findings from this synthesis highlight that the perceived inability of self-test kits to accurately test for HIV, and the misinterpretation of test results may undermine the uptake of self-test kits. Participants across studies included in this synthesis agreed that information on how rapid HIV tests function may alleviate misconceptions, thus improving the uptake of testing [22, 24].

Irrespective of existing global evidence on potential benefits of HIVST, [16, 22–25, 41, 43, 47] participants in this synthesis expressed key concerns related to HIVST, such as human rights issues, lack of regulatory and quality assurance systems, low quality of, lack of linkage to care, and face-to-face counseling. These findings align with existing literature on HIVST, whereby concerns related to HIVST were reported. For example, Johnson and colleagues, [15] agreed that state regulation was an essential requirement to achieve quality assurance, and hence promote quality of self-test kits to the advantage of the users. However, caution was raised regarding state regulation to restrict access to HIVST, such as setting an age limit for purchasing test kits, because the purchase of an HIVST kit was considered to be a personal decision that should not be interfered by the state [27, 48–50]. Therefore, it behooves HIV policymakers, and interventionists to develop country-specific HIVST regulatory and policy frameworks that focus on safety, prevention of coercive use, and effectiveness of HIVST [19, 51, 52].

To address the lack of linkage to care and face-to-face counseling, this synthesis recommends innovative counseling and training approaches for users of HIVST. Strategies to increase linkage to HIV prevention, care and treatment after HIVST include home visits or phone calls, [53] and demand-side financial incentives [54]. Strategies, such as the use of toll-free phone numbers provided by the manufacturers of HIVST for counseling have been perceived of greater quality than face-to-face counseling in different settings [55, 56].

Further research is warranted to evaluate algorithms and methods that will facilitate adequate linkage to care following HIVST [19, 51, 52]. However, Gagnon et al., [27] caution that such strategies could indirectly propagate stigma by making HIV testing a “clandestine activity” done in the home settings in secrecy.

This synthesis identified two qualitative studies, reporting self-testing experiences among adult men and women in Kenya, Malawi, and South Africa [6, 40]. Participants reported that the availability of HIVST creates an opportunity for re-initiation of ART, suggesting that defaulter may be more likely to prefer HIVST than standard HTS to re-initiate HIV care and treatment.

Among HIVST users, easy to use of self-test kits and preference of oral fluid-based HIV rapid test (RDT) because it is less invasive and painless was frequently cited across two studies included in this synthesis. These findings align with existing literature on HIVST, suggesting that most users (even with low literacy) find HIVST is easy to use, [57] and may prefer oral fluid-based HIV RDT to finger stick/ or whole blood-based HIV [6, 55, 58].

Confusion on how to use self-test kits was cited as the main cause of user errors and inaccuracy, particularly with unsupervised HIVST [6]. This observation concurs with findings among female sex workers in Uganda, [59] and underlines the need to provide training on HIVST use, accompanied with clear pictorial instruction-for-use in local language on how to perform HIVST, easy steps to interpret the test result, and linkage to support and counseling services [55, 59–61].

Further, different products of self-test kits with different manufacturer’s instruction were cited to increase rates of wrong interpretation of test results. In considering new HIVST products, which are under development and could be adapted for HIVST, caution should be made to manufacturers to develop user-friendly HIVST products to reduce the rates of user errors [22, 24].

### Strengths and limitation

The strength of this synthesis is based on the systematic search of multiple databases to identify all relevant qualitative studies meeting the predetermined inclusion criteria. Additionally, we included studies using different methodological approaches, contributing to the in-depth understanding of HIV stakeholder’s perceptions of the factors that enable or deter the uptake of HIV self-testing in Africa. Another strength is the inclusion of studies conducted among actual users of HIVST, which provides findings relevant to the research question, as opposed to studies conducted on hypothetical use of HIVST.

Studies were not excluded based on the overall ‘low quality’; so long they contributed relevant qualitative evidence, resulting in a comprehensive review capturing a range of perspectives to the study objectives. Another strength was the use of the CASP tool for methodological quality assessment, [28] and *CERQual***,** for the confidence of qualitative evidence [29]. The use of these multiple approaches, had advantages, including the possibility to reach conclusions based on similarities identified in heterogeneous studies, more accessibility to a wider audience than primary studies, and provide a weight of evidence about HIVST.

There are however some limitations of this synthesis. Foremost, the possibility that this review might not reflect all the barriers, facilitators and actual user’s experiences related to HIVST that are relevant could be a limitation because of a few studies conducted across African countries. Most primary studies reported views of HIV stakeholders about the hypothetical use of HIVST, which may not reflect their actual practice. Only two primary studies reported the actual practice of self-testing among adult users, indicating a need for further research on HIVST testing experiences across different populations in Africa. Secondly, there is the possibility of having missed some publications. To mitigate this limitation, we scanned references of selected papers for additional studies. Due to language constraints, we only included papers published in English, and the findings reported henceforth may be subject to English language publication bias.

## Conclusions

Generally, the uptake of HIV testing in Africa is a complex process influenced by multifaceted and interlinked factors. This synthesis contributed to a literature gap on HIVST by identifying important factors that enable or deter the uptake of HIVST among adults in Africa. While identified facilitators of, and barriers to the uptake of HIVST cut across studies from sub-regions of Africa, HIVST interventionists should develop context-specific, culturally appropriate strategies to increase uptake of HIV testing using HIVST. Actual HIVST users expressed preference of oral-fluid self-testing because it is easy to use, less invasive and painless compared to finger-stick/whole blood-based HIV tests. Lack of clear instructions on how to use self-test kits, and existing different products of HIVST increases rates of user errors. If adopted as a complimentary HTS option, HIVST could facilitate early detection, early care, treatment, and prevention, and maybe pivotal in providing an invaluable tool to increase access to HIV care, treatment, and prevention to achieve the 95–95-95 by 2030 [1, 2].

## Supplementary information

**Additional file 1:** Describes the literature search for PubMed, CINAHL, & Web of Science

**Additional file 2:** Data extraction form: Qualitative studies

**Additional file 3:** **Table S3.** ENTREQ checklist (Enhancing transparency in reporting the synthesis of qualitative research) *

## Data Availability

The datasets supporting the conclusions of this synthesis are included in this published article (and within the supplementary information files).

## Abbreviations

ART: Antiretroviral Treatment
CASP: Critical Appraisal Skills Programme
CERQual: Confidence in the Evidence from Reviews of Qualitative research
ENTEREQ: Enhancing transparency in reporting the synthesis of qualitative research
GRADE: Grading of Recommendations Assessment, Development and Evaluation
HIVST: HIV Self-Testing
HTS: HIV Testing Services
LMIC: Low and Middle-Income countries
RDT: Rapid Test
SSA: Sub-Saharan Africa
