# Supplementary material for: A systematic review of qualitative evidence on factors enabling and deterring uptake of HIV self-testing in Africa
Source: BMC Public Health. 2019 Oct 15;19:1289. doi: 10.1186/s12889-019-7685-1 (PMC6794839; doi:10.1186/s12889-019-7685-1)
Supplement: Supplementary file 1 — Additional file 1: Describes the literature search for PubMed, CINAHL, & Web of Science [file 12889_2019_7685_MOESM1_ESM.docx]

| **Supplementary 1: Describes the literature search for PubMed, CINAHL, & Web of Science** | | |
| --- | --- | --- |
| **Search** | **Query PubMed:** | **Items** |
| #6 | #1 AND #2 AND #3 AND #4 AND #5 | 6 |
| #5 | Search AFRICA OR (((ALGERIA) OR (ANGOLA) OR (BENIN) OR (BOTSWANA) OR (BURKINA FASO) OR (BURUNDI) OR (CAMEROON) OR ((CANARY ISLANDS) OR "CANARY ISLANDS") OR ((CAPE VERDE) OR "CAPE VERDE") OR (CENTRAL AFRICAN REPUBLIC) OR (CHAD) OR (COMOROS) OR (CONGO) OR (DEMOCRATIC REPUBLIC CONGO) OR (DJIBOUTI) OR (EGYPT) OR ((EQUATORIAL GUINEA) OR "EQUATORIAL GUINEA") OR (ERITREA) OR (ETHIOPIA) OR (GABON)) OR ((GAMBIA) OR (GHANA) OR (GUINEA) OR ((GUINEA BISSAU) OR "GUINEA BISSAU") OR (IVORY COAST) OR ((COTE D'IVOIRE) OR "COTE D'IVOIRE") OR ((COTE IVOIRE) OR "COTE IVOIRE") OR (KENYA) OR (LESOTHO) OR (LIBERIA) OR (LIBYA) OR (LIBIA) OR (JAMAHIRIYA) OR (JAMAHIRYIA) OR (MADAGASCAR) OR (MALAWI) OR (MALI) OR (MAURITANIA) OR (MAURITIUS) OR (MOROCCO)) OR ((MOZAMBIQUE) OR (MOCAMBIQUE) OR (NAMIBIA) OR (NIGER) OR (NIGERIA) OR (REUNION) OR (RWANDA) OR ((SAO TOME) OR "SAO TOME") OR (SENEGAL) OR (SEYCHELLES) OR ((SIERRA LEONE) OR "SIERRA LEONE") OR (SOMALIA) OR ((SOUTH AFRICA) OR "SOUTH AFRICA") OR ((ST HELENA) OR "ST HELENA") OR (SUDAN) OR (SWAZILAND) OR (TANZANIA) OR (TANGANYIKA) OR (TOGO) OR (TUNISIA)) OR ((UGANDA) OR ((WESTERN SAHARA) OR "WESTERN SAHARA") OR (ZAIRE) OR (ZAMBIA) OR (ZIMBABWE) OR (AFRICA[MH]) OR (SOUTH* AND AFRICA*) OR (WEST* AND AFRICA*) OR (EAST* AND AFRICA*) OR (NORTH* AND AFRICA*) OR (CENTRAL* AND AFRICA*) OR (SUB SAHARAN AFRICA*) OR (SUBSAHARAN AFRICA*) OR (AFRICA*))) NOT (((GUINEA PIG*) OR "GUINEA PIG*") OR ((ASPERGILLUS NIGER) OR "ASPERGILLUS NIGER")) | 990,129 |
| #4 | Search Facilitat* OR Motivat* | 5,917 |
| #3 | Search Barrier* OR challeng* OR obstacle* OR Imped* OR experience* OR Perspective* OR Perception* | 1,595,831 |
| #2 | Search HIV test* OR home test* OR (self-test*) OR (HIV self-testing) OR (unsupervised self-testing) OR (supervised self-testing) | 80,265 |
| #1 | Search ("adult"[MeSH Terms] OR "adult"[All Fields] OR "adolescent"[MeSH] Terms] OR "adolescent"[All Fields] OR "young adult"[MeSH Terms] OR "adult"[MeSH Terms:noexp] OR "middle aged"[MeSH Terms] OR "aged"[MeSH Terms] OR "aged, 80 and over"[MeSH Terms]) | 7,573,014 |

| **Search** | **Query CINAHL:** | **Items** |
| --- | --- | --- |
| #6 | #1 AND #2 AND #3 AND #4 AND #5 | 222 |
| #5 | Search AFRICA OR (((ALGERIA) OR (ANGOLA) OR (BENIN) OR (BOTSWANA) OR (BURKINA FASO) OR (BURUNDI) OR (CAMEROON) OR ((CANARY ISLANDS) OR "CANARY ISLANDS") OR ((CAPE VERDE) OR "CAPE VERDE") OR (CENTRAL AFRICAN REPUBLIC) OR (CHAD) OR (COMOROS) OR (CONGO) OR (DEMOCRATIC REPUBLIC CONGO) OR (DJIBOUTI) OR (EGYPT) OR ((EQUATORIAL GUINEA) OR "EQUATORIAL GUINEA") OR (ERITREA) OR (ETHIOPIA) OR (GABON)) OR ((GAMBIA) OR (GHANA) OR (GUINEA) OR ((GUINEA BISSAU) OR "GUINEA BISSAU") OR (IVORY COAST) OR ((COTE D'IVOIRE) OR "COTE D'IVOIRE") OR ((COTE IVOIRE) OR "COTE IVOIRE") OR (KENYA) OR (LESOTHO) OR (LIBERIA) OR (LIBYA) OR (LIBIA) OR (JAMAHIRIYA) OR (JAMAHIRYIA) OR (MADAGASCAR) OR (MALAWI) OR (MALI) OR (MAURITANIA) OR (MAURITIUS) OR (MOROCCO)) OR ((MOZAMBIQUE) OR (MOCAMBIQUE) OR (NAMIBIA) OR (NIGER) OR (NIGERIA) OR (REUNION) OR (RWANDA) OR ((SAO TOME) OR "SAO TOME") OR (SENEGAL) OR (SEYCHELLES) OR ((SIERRA LEONE) OR "SIERRA LEONE") OR (SOMALIA) OR ((SOUTH AFRICA) OR "SOUTH AFRICA") OR ((ST HELENA) OR "ST HELENA") OR (SUDAN) OR (SWAZILAND) OR (TANZANIA) OR (TANGANYIKA) OR (TOGO) OR (TUNISIA)) OR ((UGANDA) OR ((WESTERN SAHARA) OR "WESTERN SAHARA") OR (ZAIRE) OR (ZAMBIA) OR (ZIMBABWE) OR (AFRICA[MH]) OR (SOUTH* AND AFRICA*) OR (WEST* AND AFRICA*) OR (EAST* AND AFRICA*) OR (NORTH* AND AFRICA*) OR (CENTRAL* AND AFRICA*) OR (SUB SAHARAN AFRICA*) OR (SUBSAHARAN AFRICA*) OR (AFRICA*))) NOT (((GUINEA PIG*) OR "GUINEA PIG*") OR ((ASPERGILLUS NIGER) OR "ASPERGILLUS NIGER")) | 49,020 |
| #4 | Search Facilitat* OR Motivat* |  |
| #3 | Search Barrier* OR challeng* OR obstacle* OR Imped* OR experience* OR Perspective* OR Perception* | 462,170 |
| #2 | Search HIV test* OR home test* OR (self-test*) OR (HIV self-testing) OR (unsupervised self-testing) OR (supervised self-testing) | 8,099 |
| #1 | Search ("adult"[MeSH Terms] OR "adult"[All Fields] OR "adolescent"[MeSH] Terms] OR "adolescent"[All Fields] OR "young adult"[MeSH Terms] OR "adult"[MeSH Terms:noexp] OR "middle aged"[MeSH Terms] OR "aged"[MeSH Terms] OR "aged, 80 and over"[MeSH Terms]) | 838,517 |

| **Search** | **Query Web of Science:** | **Items** |
| --- | --- | --- |
| #6 | #1 AND #2 AND #3 AND #4 AND #5 | 24 |
| #5 | Search AFRICA OR (((ALGERIA) OR (ANGOLA) OR (BENIN) OR (BOTSWANA) OR (BURKINA FASO) OR (BURUNDI) OR (CAMEROON) OR ((CANARY ISLANDS) OR "CANARY ISLANDS") OR ((CAPE VERDE) OR "CAPE VERDE") OR (CENTRAL AFRICAN REPUBLIC) OR (CHAD) OR (COMOROS) OR (CONGO) OR (DEMOCRATIC REPUBLIC CONGO) OR (DJIBOUTI) OR (EGYPT) OR ((EQUATORIAL GUINEA) OR "EQUATORIAL GUINEA") OR (ERITREA) OR (ETHIOPIA) OR (GABON)) OR ((GAMBIA) OR (GHANA) OR (GUINEA) OR ((GUINEA BISSAU) OR "GUINEA BISSAU") OR (IVORY COAST) OR ((COTE D'IVOIRE) OR "COTE D'IVOIRE") OR ((COTE IVOIRE) OR "COTE IVOIRE") OR (KENYA) OR (LESOTHO) OR (LIBERIA) OR (LIBYA) OR (LIBIA) OR (JAMAHIRIYA) OR (JAMAHIRYIA) OR (MADAGASCAR) OR (MALAWI) OR (MALI) OR (MAURITANIA) OR (MAURITIUS) OR (MOROCCO)) OR ((MOZAMBIQUE) OR (MOCAMBIQUE) OR (NAMIBIA) OR (NIGER) OR (NIGERIA) OR (REUNION) OR (RWANDA) OR ((SAO TOME) OR "SAO TOME") OR (SENEGAL) OR (SEYCHELLES) OR ((SIERRA LEONE) OR "SIERRA LEONE") OR (SOMALIA) OR ((SOUTH AFRICA) OR "SOUTH AFRICA") OR ((ST HELENA) OR "ST HELENA") OR (SUDAN) OR (SWAZILAND) OR (TANZANIA) OR (TANGANYIKA) OR (TOGO) OR (TUNISIA)) OR ((UGANDA) OR ((WESTERN SAHARA) OR "WESTERN SAHARA") OR (ZAIRE) OR (ZAMBIA) OR (ZIMBABWE) OR (AFRICA[MH]) OR (SOUTH* AND AFRICA*) OR (WEST* AND AFRICA*) OR (EAST* AND AFRICA*) OR (NORTH* AND AFRICA*) OR (CENTRAL* AND AFRICA*) OR (SUB SAHARAN AFRICA*) OR (SUBSAHARAN AFRICA*) OR (AFRICA*))) NOT (((GUINEA PIG*) OR "GUINEA PIG*") OR ((ASPERGILLUS NIGER) OR "ASPERGILLUS NIGER")) | 74 |
| #4 | Search Facilitat* OR Motivat* | 291 |
| #3 | Search Barrier* OR challeng* OR obstacle* OR Imped* OR experience* OR Perspective* OR Perception* | 2,743 |
| #2 | Search HIV test* OR home test* OR (self-test*) OR (HIV self-testing) OR (unsupervised self-testing) OR (supervised self-testing) | 11,340 |
| #1 | Search ("adult"[MeSH Terms] OR "adult"[All Fields] OR "adolescent"[MeSH] Terms] OR "adolescent"[All Fields] OR "young adult"[MeSH Terms] OR "adult"[MeSH Terms:noexp] OR "middle aged"[MeSH Terms] OR "aged"[MeSH Terms] OR "aged, 80 and over"[MeSH Terms]) | 61,241 |
