# Supplementary material for: A systematic review of qualitative evidence on factors enabling and deterring uptake of HIV self-testing in Africa
Source: BMC Public Health. 2019 Oct 15;19:1289. doi: 10.1186/s12889-019-7685-1 (PMC6794839; doi:10.1186/s12889-019-7685-1)
Supplement: Supplementary file 2 — Additional file 2: Data extraction form: Qualitative studies [file 12889_2019_7685_MOESM2_ESM.docx]

| **Supplementary 2. Data extraction form: Qualitative studies** | | | | |
| --- | --- | --- | --- | --- |
| Title: Factors that influence the uptake of HIV self-testing, and testing experiences of adult users in Africa. | | | | |
| Date form completed[dd/mm/yyyy] | | | | _ _/_ _/_ _ _ _ |
| **A: Source** | | | | |
| Reviewer ID: | |  | | |
| Study ID (surname of first author and year of first full report of study was published | | | | |
| Title |  | | | |
| Authors |  | | | |
| Contact details |  | | | |
| Published | Yes |  | No |  |
| If yes, please provide citation/ year of study/publication/ name of journal/language. |  | | | |
| Reference of potentially eligible studies from the reference list. |  | | | |
| **B: Eligibility** | | | | |
| Confirm eligibility for review | Yes |  | No |  |
| Insert inclusion criteria for each characteristic as defined in the protocol | | | | |
| Characteristics | Page | Figure | Table | Other |
|  |  |  |  |  |
| Decision taken | **INCLUDE** |  | **EXCLUDE** |  |
| If not, give reason(s) for exclusion |  | | | |
| **DO NOT PROCEED IF STUDY EXCLUDED FROM REVIEW** | | | | |
| **C: Qualitative study characteristics** | | | | |
| **Characteristics** | **Description as stated in article/paper** | | **Location in text or source** | |
| Overall aim/purpose |  |  |  |  |
| Research/analytical question(s) |  |  |  |  |
| Methodology |  |  |  |  |
| Geographical setting(s) |  |  |  |  |
| Cultural setting/social context |  |  |  |  |
| Participants |  |  |  |  |
| **Data collection method:** | | | | |
| Direct observation |  |  |  |  |
| In-depth interviews |  |  |  |  |
| Focus group discussion |  |  |  |  |
| Group discussion |  |  |  |  |
| Other(s) |  |  |  |  |
| Outcome(s) |  |  |  |  |
| Methods of synthesis |  |  |  |  |
| **Checklist for qualitative studies** | | | | |
| Type of qualitative study | Description and assessment | | Comments | Location in text/source |
|  | Participant observation |  |  |  |
|  | Open-ended interviews |  |  |  |
|  | Structured interviews |  |  |  |
|  | Others(specify) |  | | |
| **Theoretical approach** | Appropriate | Inappropriate | Not sure |  |
| Ethical consideration | | | | |
| Was the study approved by an ethics committee (i.e., is ethics approval number available?) | | | | |
| Other relevant information |  | | | |
| Key study findings |  | | | |
| Notes |  | | | |
