# Supplementary material for: A systematic review of qualitative evidence on factors enabling and deterring uptake of HIV self-testing in Africa
Source: BMC Public Health. 2019 Oct 15;19:1289. doi: 10.1186/s12889-019-7685-1 (PMC6794839; doi:10.1186/s12889-019-7685-1)
Supplement: Supplementary file 3 — Additional file 3: Table S3. ENTREQ checklist (Enhancing transparency in reporting the synthesis of qualitative research) * [file 12889_2019_7685_MOESM3_ESM.docx]

S 3. Table: ENTREQ checklist (Enhancing transparency in reporting the synthesis of qualitative research) *

| **No. Item** | **Guide Questions/Description** | **Reported on Page** |
| --- | --- | --- |
| 1. Aim | The aim was to synthesize the evidence from qualitative studies of HIV stakeholder’s perceptions about factors that facilitate or hinder the uptake of HIV self-testing (HIVST), and experiences of HIV self-testing of adult users in Africa. | Page 2. |
| 2. Synthesis methodology | Identify the synthesis methodology or theoretical framework, which underpins the synthesis, and describe the rationale for choice of methodology (e.g. thematic synthesis). | Page 7. |
| 3. Approach to  searching | The search was pre-planned. Comprehensive search strategies were undertaken to seek all available studies. | Page 5. |
| 4. Inclusion criteria | Included studies used widely accepted qualitative data collection methods, with well-described methodology, including for example: interviews, focus groups, direct observation, and participatory action research. Included studies also needed to have provided a clear description of recognized qualitative data analysis methods (e.g., grounded theory, narrative analysis, content analysis, thematic analysis).  Excluded studies included those for which it was difficult to extract qualitative data, e.g., mixed methods studies without clearly labeled data, or studies in settings where perceptions of HIV stakeholders or HIVST potential and/ or actual users could not be clearly identified, such as summaries or aggregated data. Commentaries, protocols, and systematic reviews were not included in the analysis. Additionally, as the focus was on research from Africa, studies from countries outside Africa were excluded. | Page 5. |
| 5. Data sources | The following electronic databases were considered to be the most relevant for the topic and were searched: CINAHL, MEDLINE in PubMed, and Web of Science electronic databases using guidelines developed by the Cochrane Qualitative Research Methods Groups for searching for qualitative evidence. All data were limited to publications in English from 1998 to 2018. | Page 5. |
| 6. Electronic Search strategy | Additional file S. 1 describes the literature search for PubMed, CINAHL, and Web of science. | Supplementary # 1 |
| 7. Study screening methods | Two independent reviewers screened study titles and abstracts for suitability against inclusion and exclusion criteria. The decision to include or exclude a study was required to be agreed on by both reviewers. If after consultation the two reviewers didn’t reach a decision, a third reviewer made the final decision. | Page 5 & Figure 1. |
| 8. Study characteristics | Table 1 presents the characteristics of the included studies (author(s), year of study, country, study aims, study design and methods, population/ number of participants, and summary of findings). | Table 1. |
| 9. Study selection  results | A flow diagram using PRISMA guidelines for reporting of systematic reviews is presented in Figure 1 in reporting of the selection process and results. We identified 258 records from three search engines. 232 records were screened after removing duplicates and excluded 174 records. 58 full-text articles were assessed for eligibility, and 49 articles were excluded for the following reasons: 1) outcomes not of interest (n=22); 2) unclear study design (n=18); and 3) participants not of interest/studies outside Africa. As a result, we included 9 studies in the qualitative synthesis. | Page 5 & Figure 1. |
| 10. Rationale for  appraisal | Critical Appraisal Skills Program (CASP) guidelines and GRADE- CERQual were used to assess qualitative evidence syntheses findings. CASP offers a valid checklist to help researchers appraise and understand qualitative studies better. GRADE-CERQual helps researchers assess how much confidence to place in findings from a qualitative evidence synthesis. First, each selected document was initially assessed for quality and internal validity according to the CASP checklist for qualitative research. The CASP checklist includes 10 questions to appraise the quality of qualitative research. These assessments for each study can be seen in Table 2. Selected studies met minimum criteria defined through the checklist including domains such as appropriateness of study design, data collection techniques, and analysis methods used. At the second level of appraisal, the GRADE-CERQual guidance was used to differentiate emergent findings strongly supported or less well supported. Two reviewers independently reviewed studies using guidance derived from GRADE-CERQual to reach consensus of the quality of findings emergent from included studies. The results of this appraisal are presented in Table 3. | Page 6-7; Table 2 & Table 3. |
| 11. Appraisal items | CASP guidelines and GRADE-CERQual were used to assess qualitative evidence syntheses findings. | Page 6-7. |
| 12. Appraisal process | Two independent reviewers conducted appraisal independently. The reviewers discussed if consensus was required. | Page 6. |
| 13. Appraisal results | Appraisal results are presented in Tables 2 and 3 | Tables 2 & 3. |
| 14. Data extraction | For organization of extracted data, a unified matrix was utilized to record specific characteristics of included studies. Extracted data included: reference details (author, year, title, journal/publisher); country/region of study; objectives or aims of the study; study design including methodological approaches (e.g., interviews/focus groups) and conceptual basis underlying the study (e.g., Grounded Theory); analysis method(s); sampling methodology and sample size; and initial assessment of the methodological limitations of the study. The results of the selection process and data abstraction are presented in Figure 1 & Table 1. | Page 5; Figure 1, Table 1 & Supplementary # 2. |
| 5. Software | Qualitative software was not used. | N/A |
| 16. Number of reviewers | Two reviewers were involved in coding and analysis. | Page 9. |
| 17. Coding | Thematic analysis was employed to identify domains descriptive of the data for investigation and presentation. | Page 9. |
| 18. Study comparison | Similar findings were coded into key themes and categories within and across studies. GRADE-CERQual was used to assess confidence in qualitative evidence syntheses findings across studies. | Page 6-9; Table 3. |
| 19. Derivation of themes | The process of deriving the themes and categories were inductive to assess previously researched phenomena. | Page 6. |
| 20. Quotations | Findings and quotations from the primary studies to illustrate themes and constructs, are presented in the result section. | Page 10-18. |
| 21. Synthesis output | Synthesis output is presented in Table 3. In this synthesis, twenty-one (21) statements were generated and summarized into four (4) themes: potential facilitators of HIVST, perceived barriers to HIVST, concern about HIVST, and HIVST experiences. Through this qualitative synthesis, potential facilitators of HIVST perceived barriers to HIVST, and concerns about HIVST were identified. The synthesis also focused on identifying studies that related the experiences of actual HIVST adult users in Africa. The findings presented in this synthesis underscored the complexity in addressing HIV testing, and HIVST in particular, and its associated challenges. . The findings presented in this review have important implications for HIV stakeholders, HIVST users, and public health in general. | Page 10-23. |

* Reference: Tong A, Flemming K, McInnes E, Oliver SA, Craig J. Enhancing transparency in reporting the synthesis of qualitative research: ENTREQ. BMC Medical Research Methodology 2012, 12:181.
